# Comparative Evaluation of Masticatory Bite Force of Implant-Supported Overdenture With Different Attachments: An In Vivo Study

**DOI:** 10.7759/cureus.96834

**Published:** 2025-11-14

**Authors:** Pallavi Mundra, Rajeev Srivastava, Raveena Makker, Sourabh Khandelwal, Santosh K Bung, Sonu Solanki

**Affiliations:** 1 Prosthodontics, Index Institute of Dental Sciences, Indore, IND; 2 Prosthodontics and Crown & Bridge, Index Institute of Dental Sciences, Indore, IND; 3 Prosthodontics, Navodaya Dental College, Raichur, IND

**Keywords:** attachment system, bite force, chewing efficiency, fujifilm pressure measuring sheet, implants, masticatory bite force, overdentures, prescale system, resorbed ridges

## Abstract

Statement of problem: Research assessing the bite force generated by implant-supported overdentures using various attachments.

Purpose: This in vivo study aimed to examine the masticatory bite force of various attachments utilized in implant-supported overdentures.

Materials and methods: Eight completely edentulous patients with two mandibular implants were selected for the creation of a mandibular implant-supported overdenture. The processes for denture fabrication were executed traditionally to produce one maxillary denture and two mandibular dentures. The mandibular dentures were subsequently transformed into overdentures using ball and flat attachments, respectively, through the pickup method. Masticatory bite force was measured with both attachments on the left and right sides at the time of denture insertion, as well as after one month of wearing the dentures, using a bite force measuring device.

Result: Data were analyzed with the Statistical Package for Social Sciences version 21.0 (IBM Corp., Armonk, NY, USA). Normality distribution was assessed using the Kolmogorov-Smirnov test, which showed a normal distribution. Consequently, a parametric test of significance was used. Descriptive statistics were carried out, and data were expressed as mean and standard deviation. Comparisons between groups were conducted using the independent t-test, while intra-group comparisons were conducted using the paired t-test. A p-value of less than 0.5 was deemed statistically significant.

Conclusion: The study concluded that masticatory bite force was higher with flat attachments than with ball attachments. Additionally, the force increased after one month of insertion.

## Introduction

Edentulism refers to the condition of being without natural teeth [1]. The loss of teeth impacts a person’s function, esthetics, and health, diminishing the effectiveness of the chewing muscles and compromising nutrition, which increases vulnerability to various diseases. Therefore, it is a prevalent health issue that warrants attention [2]. Complete edentulism is associated with reduced masticatory function and unfavorable esthetics resulting from the loss of support for facial musculature, a decreased vertical dimension, and impaired speech. Patients with significantly resorbed mandibles frequently experience challenges with conventional complete dentures, particularly mandibular dentures [3]. The instability of these dentures results in ineffective chewing and general dissatisfaction with the prosthesis [4].

Dental implants are a widely used solution for replacing missing teeth, with proven long-term success and survival rates exceeding 90% [5]. Recent developments in dentistry have transitioned from a preference for traditional complete dentures to implant-supported overdentures for completely edentulous individuals. Implant-supported overdentures offer numerous benefits compared to conventional dentures, including enhanced chewing efficiency, greater masticatory bite force, and improved patient satisfaction.

According to the McGill consensus statement of 2002, using a conventional denture for patients with a completely edentulous mandible should no longer be the first-choice treatment. Instead, placing two implants and creating an implant-retained overdenture should be the preferred option [6]. Various attachment systems with diverse retentive properties have been developed for implant-supported overdentures. An ideal overdenture attachment should possess specific features to minimize clinical complications. Such an attachment allows for movement during function or removal from the mouth. Typically, plastic or silicone components engage metal parts that connect to bars or implants in the mouth [7]. The straightforward nature of attachment systems in implants promotes widespread use, especially with mandibular implant overdentures. These systems include ball, magnetic, and flat attachments [8].

Mastication marks the initial phase of digestion; it is a crucial step for nutrient intake and essential for human health and well-being [9]. A key indicator of the masticatory system's functionality is the generated bite force [10]. Bite force refers to the total force exerted by the masticatory muscles during dental occlusion and can be measured to assess the performance of the masticatory system. In dental research, bite force serves as a variable to evaluate the effectiveness of various dental treatments [11].

Aim

To evaluate and compare the masticatory bite force of implant-supported overdentures with different attachments.

Objectives

1. To evaluate the masticatory bite force of implant-supported overdentures with ball attachments (Group I).

2. To evaluate the masticatory bite force of implant-supported overdentures with flat attachments (Group II).

3. To compare the masticatory bite force of implant-supported overdenture with ball and flat attachments (Group III).

## Materials and methods

Human participants were used in this study. The study was presented before the Institutional Ethical Committee and also considered the recommendations of the Scientific Review Committee at the Index Institute of Dental Sciences, Indore (M.P). This comparative clinical study was then approved by the Institutional Ethical Committee with its respective ethical approval number as IIDS/IEC/2021/519 on 27th February 2021. This study was supposed to be completed within the duration of two years.

Inclusion criteria 

Edentulism: Completely edentulous patients requiring mandibular overdentures.

Implant placement: Patients receiving at least two implants for overdenture support.·

Attachment systems: Inclusion of patients receiving either ball or flat attachments for implant-supported overdentures.

Follow-up capability: Patients willing and able to attend follow-up visits (during insertion and after one month of insertion).

Informed consent: Patients providing informed consent for study participation.

Exclusion criteria

Systemic conditions: Patients with uncontrolled diabetes, severe osteoporosis, or other systemic diseases impacting osseointegration.

Radiation therapy: History of head/neck radiation affecting bone healing.

Active infection: Presence of active oral infection/inflammation at implant sites.

Non-cooperative patients: Patients unlikely to comply with follow-up/protocol.

Previous implant failure: History of failed implants in the same arch.

Neurological disorders: Conditions impairing the patient's ability to assess/function with a prosthesis (e.g., severe dementia).

Sample size estimation

The sample size for the present study was calculated using G*Power 3.1.9.7 software (Franz Faul Universität, Kiel, Germany), a widely recognized and reliable tool for statistical power analysis. For a t-test comparing the means of two independent groups (one-tailed), an effect size (d) of 1.43, an α error probability of 0.05, and a power (1-β) of 0.80 were used. Assuming equal allocation between groups (II/1 = 1), the analysis produced a non-centrality parameter (δ) of 2.675, a critical t-value of 1.782, and degrees of freedom (df) = 12, resulting in a required sample size of seven subjects per group (total = 14) to achieve an actual power of 0.809. To enhance the reliability and ensure adequate representation, the final sample size for the present study was rounded up to eight subjects per group, thereby exceeding the minimum requirement suggested by the power analysis. Sample size was determined based on the study by Elsyad and Khairallah [12].

Methodology 

**Figure 1:**
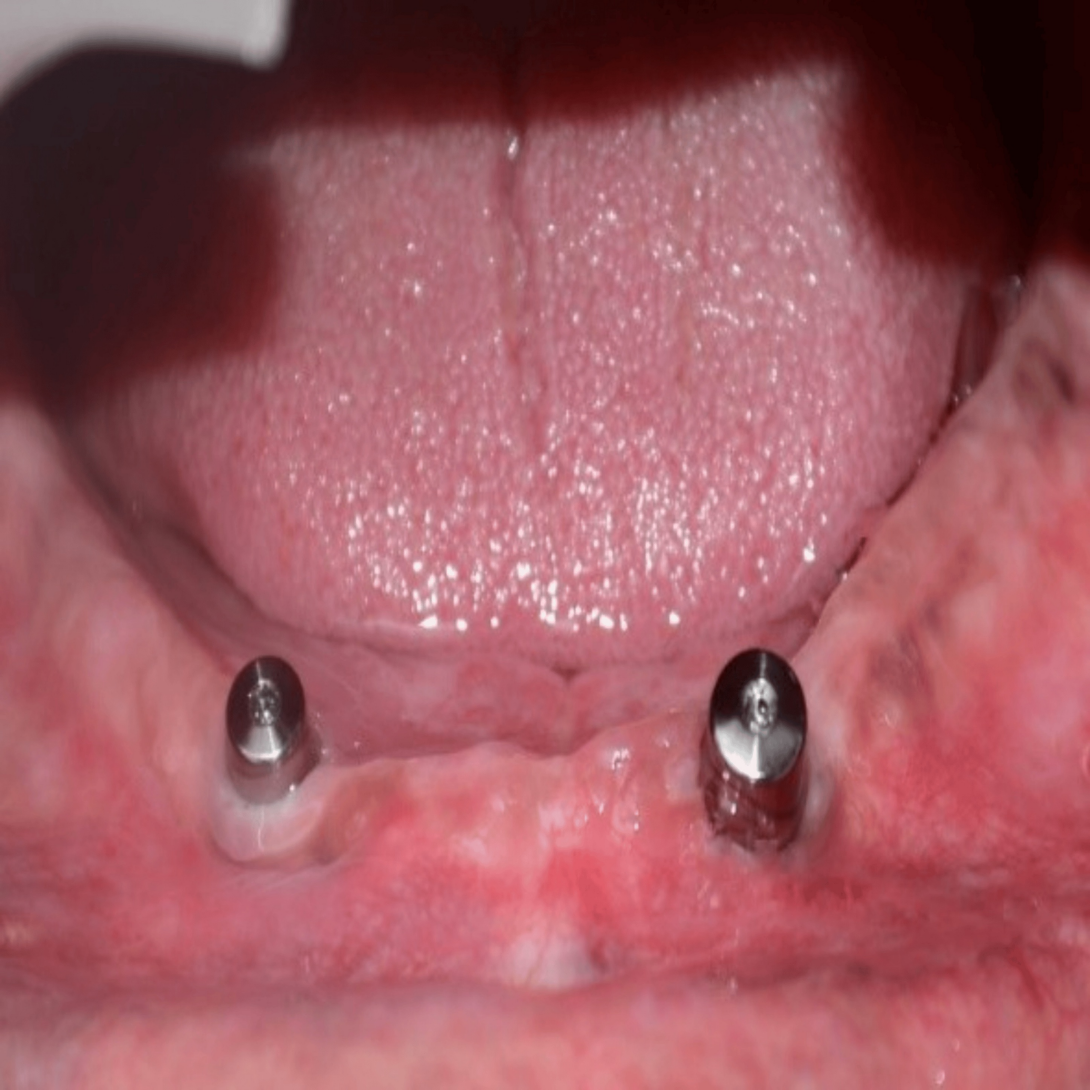
Second-stage surgery Gingival formers were placed in the second-stage surgery.

All participants provided informed consent. A comprehensive case history of all patients was recorded, after which they were recommended to undergo an orthopantomogram. Eight completely edentulous patients, each with two mandibular implants, were selected for this study. The procedure started with two small crestal incisions at the implant sites. After the implants were exposed, cover screws were replaced with gingival formers, and the second-stage surgery was completed. The prosthetic procedure commenced following the healing of the second-stage surgery, as illustrated in Figure 1.

**Figure 2:**
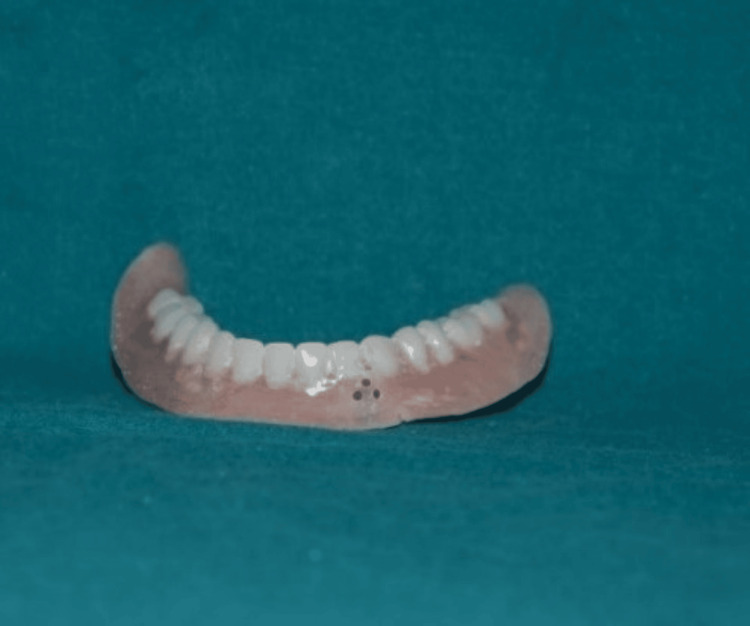
Space created for attachments in mandibular denture For ball and flat attachments, space was created in dentures.

A primary impression of the completely edentulous maxillary and mandibular arches was made using impression compound and then poured with dental plaster to get primary casts for both the maxilla and mandible. A wax spacer was adapted to the primary cast and covered with tin foil, after which a separating medium was applied on all the casts, and a special tray was fabricated from cold-cure acrylic resin. Border molding was performed using a green stick compound on all three trays. The final impression of the maxillary ridge was taken with zinc oxide eugenol impression material, while that of the mandibular ridge was done with polyvinyl siloxane impression material. The final impressions were then poured with dental stone to get master casts. As two final casts were needed for two different attachment systems (ball and flat attachments), the mandibular master cast was duplicated using a reversible hydrocolloid impression material (agar-agar). The denture base was fabricated for three master casts using cold-cure acrylic resin, followed by the making of occlusal rims. A facebow record was taken, and jaw relations were recorded and mounted on an articulator. After programming the articulator, the teeth were arranged on one maxillary and two mandibular record bases. A trial procedure was conducted. The flasking and processing steps were completed, resulting in three finished dentures. Space was created in both mandibular dentures for attachments, as depicted in Figure 2.

Placement of Attachments in a Denture

**Figure 3:**
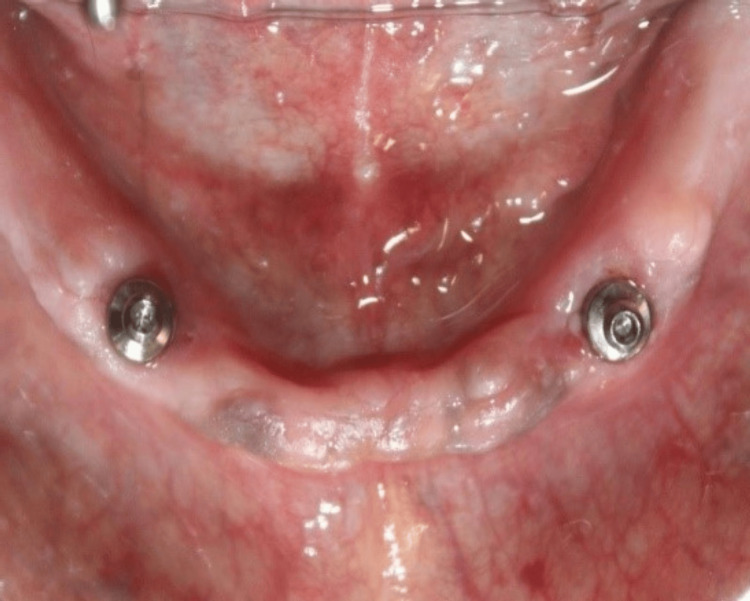
Placement of ball attachments Ball attachments were placed for an implant-supported overdenture.

The gingival formers were taken off the implants, and, as illustrated in Figure 3, ball attachments with metal housing were inserted into the implants. Next, the first denture was positioned over the mandibular ridge, which housed the ball attachments and metal housings. When the denture was removed, the metal housings were captured in the void on the intaglio surface of the denture and secured with the self-cure acrylic resin.

**Figure 4:**
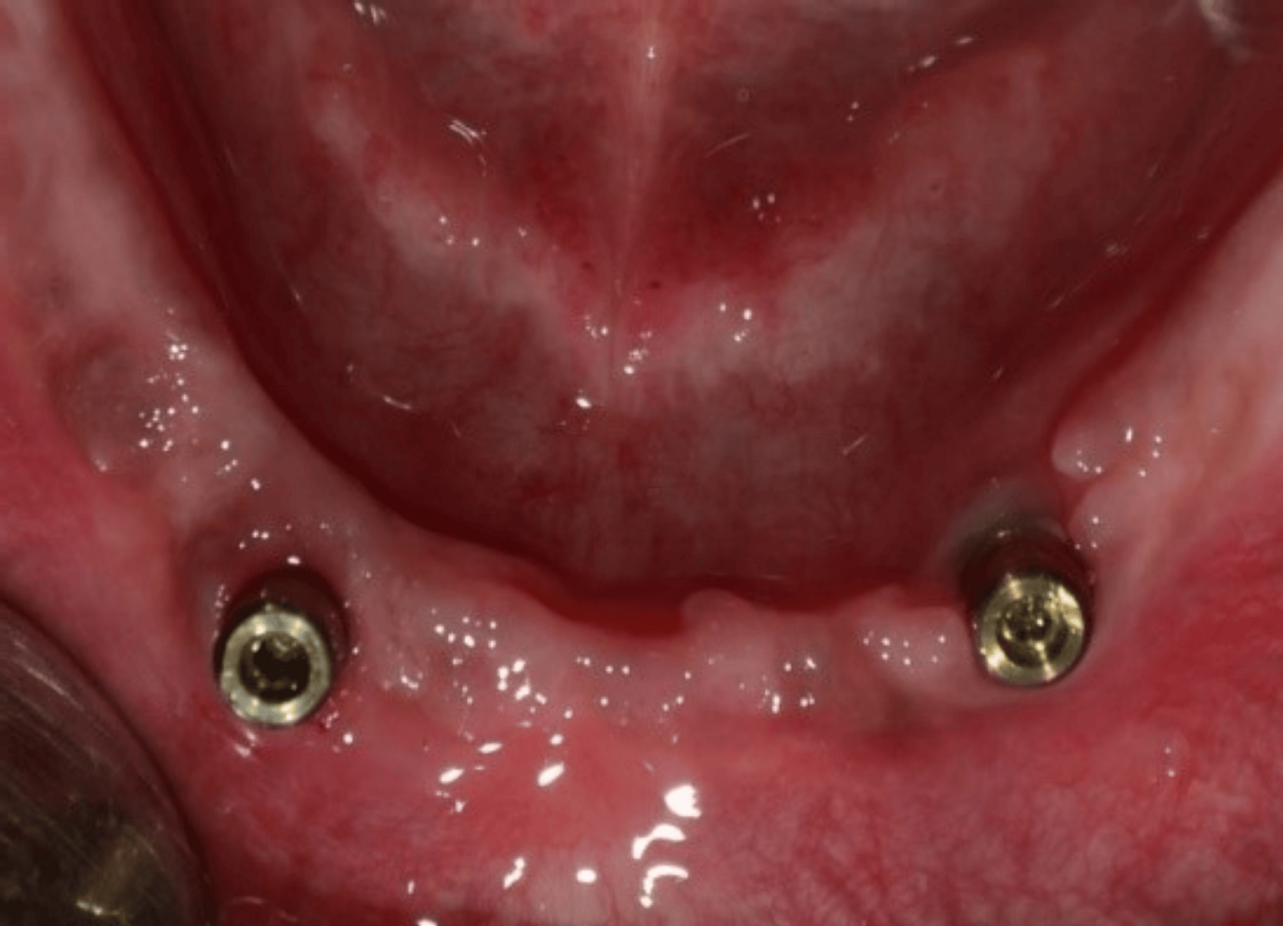
Placement of flat attachments Flat attachments were placed for an implant-supported overdenture.

Ball attachments were replaced with flat attachments in the mouth, as illustrated in Figure 4. Metal housings were then affixed to the flat attachments. The second mandibular denture was positioned onto the ridge containing flat attachments with metal housings.

**Figure 5:**
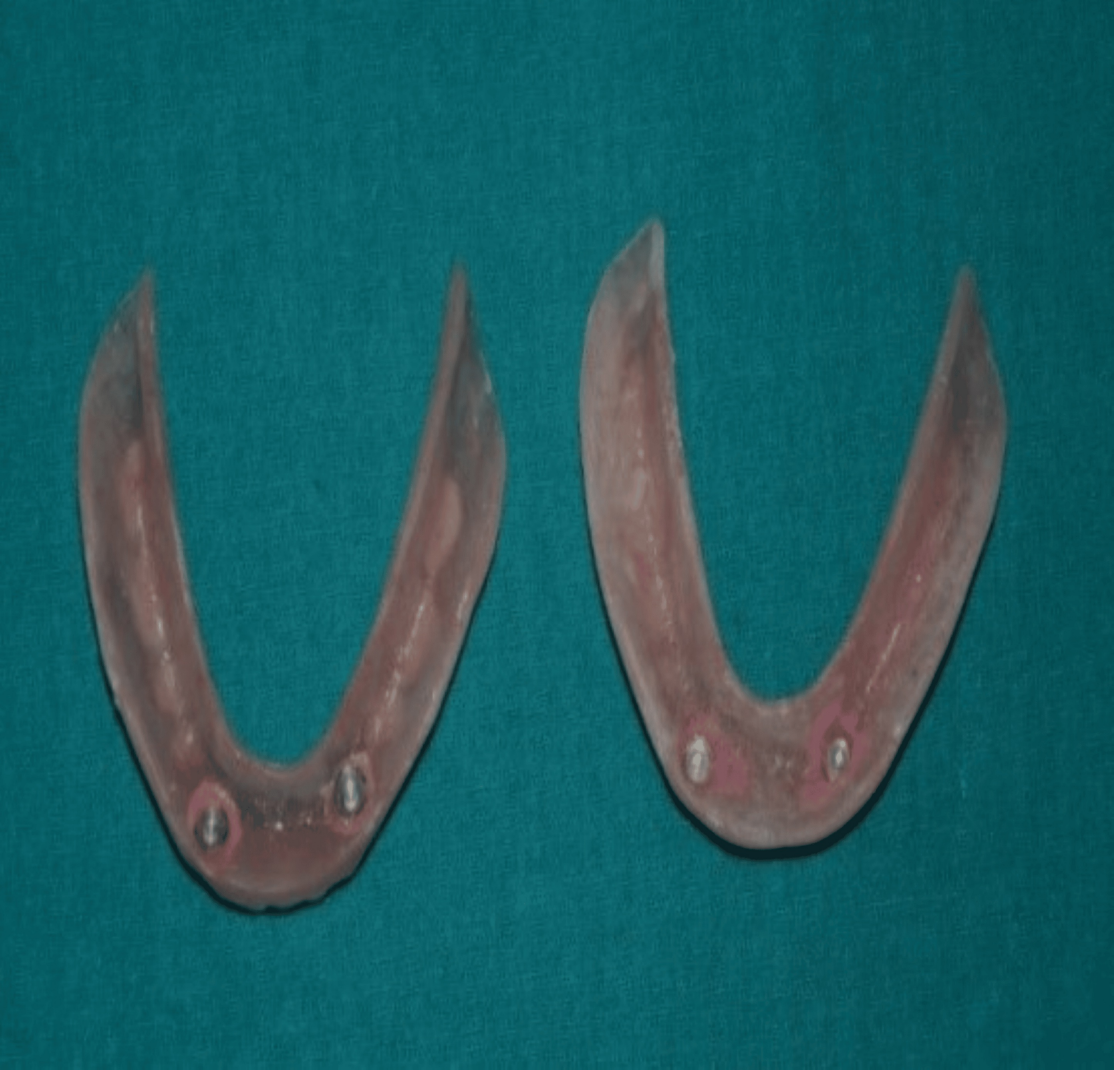
Pickup impressions Mandibular dentures with metal housing for attachments.

After the denture insertion, the patient was explained about the device. Upon removing the denture, the metal housings were retained in the space created on the intaglio surface of the denture and secured with the self-curing acrylic resin, as shown in Figure 5. 

Bite force was measured using a film on both the left and right sides.

The Bite Force Measuring Device

The bite force measuring device used in this study is the Fujifilm pressure distribution mapping system for prescale, FPD-8010e (version 2.5), manufactured by Fujifilm India Private Limited. This system scans the pressurized test piece sampled with the prescale pressure-sensitive film and displays it on the screen in full color. It enables the acquisition of various pressure-related information that was previously unattainable.

The components of the device are (1) scanner, (2) calibration sheet, (3) FPD-8010E dedicated metal cover and pad, (4) Fujifilm pressure distribution mapping system software, (5) installation manual with version information, and (6) end-user license agreement.

**Figure 6:**
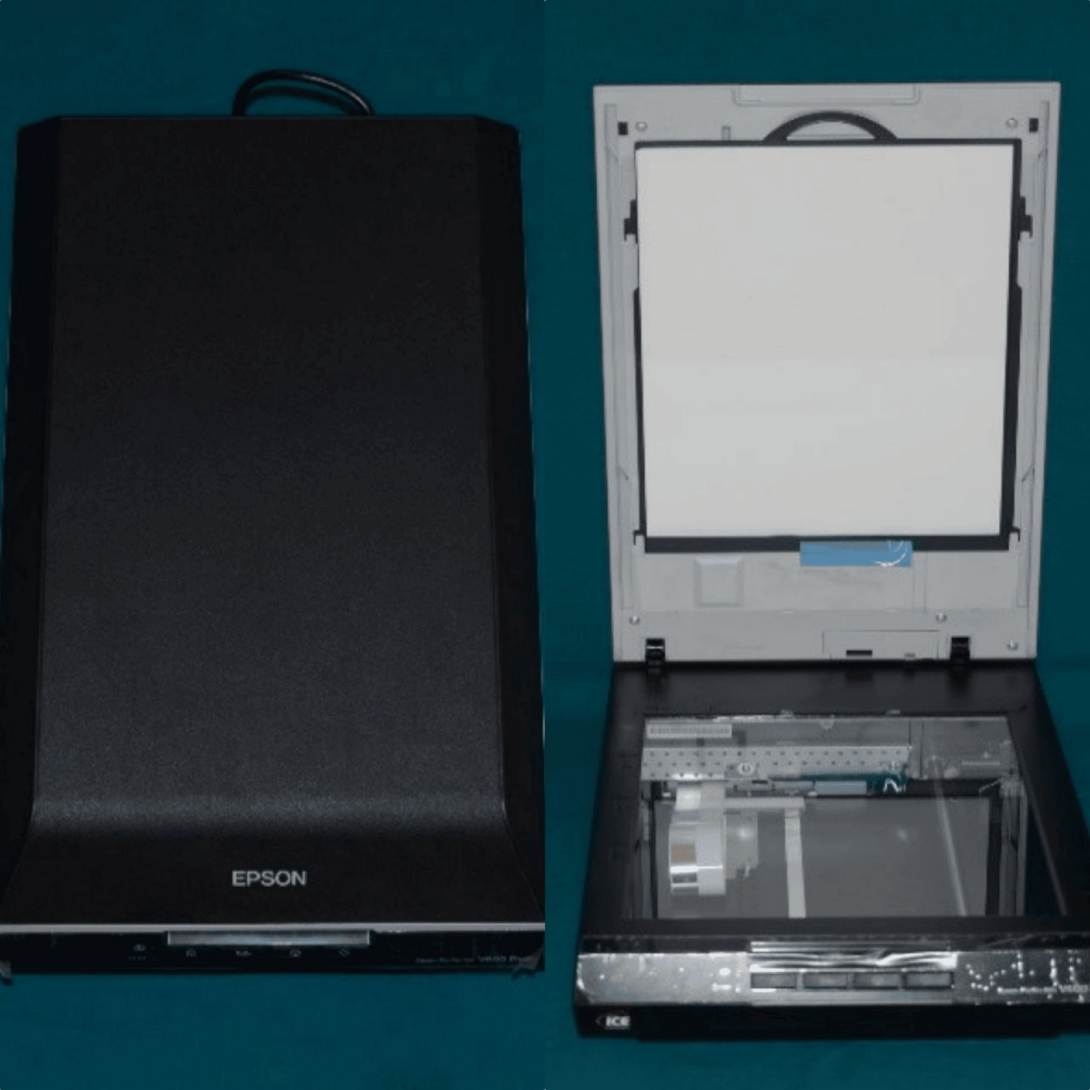
Epson Photo Scanner (Epson model V600 Perfection) This is manufactured by Seiko Epson Corporation. EPSON^®^ is a registered trademark, and Epson Perfection™ is the trademark of Seiko Epson Corporation (Suwa, Nagano, Japan).

Usage objectives: The FPD-8010E is a pressure imaging system that utilizes the Epson Photo Scanner (Epson model V600 Perfection) manufactured by Seiko Epson Corporation, to scan within the pressurization area, translating this data into pressure values as displayed in Figure 6.

**Figure 7:**
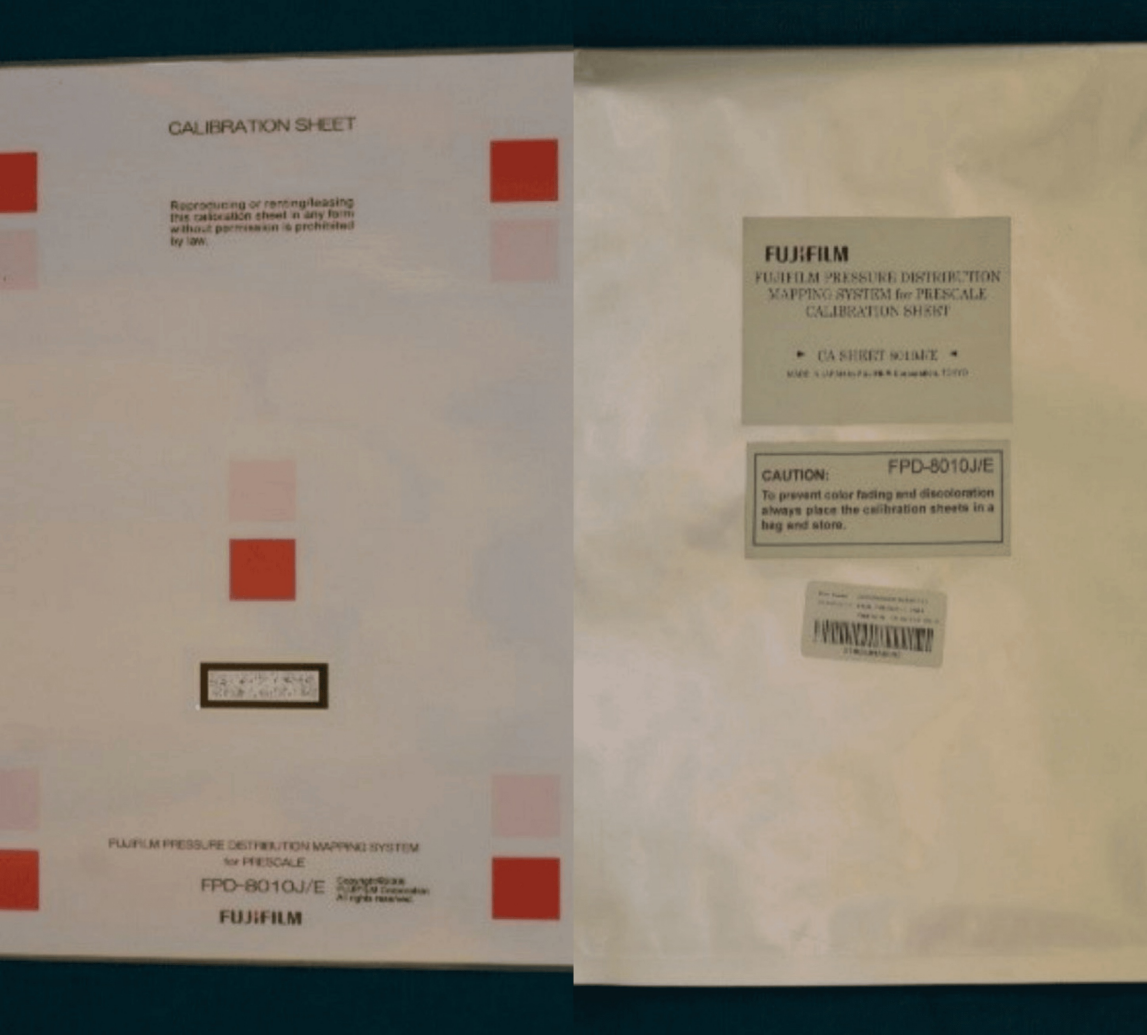
Fujifilm pressure mapping system for prescale - Calibration sheet and its cover The system comprises the calibration sheet for automatic calibration and the software, and uses the customer's computer as the processing engine. Manufacturer: Fujifilm India Private Limited (Gurugram, Haryana, India).

This system includes the Fujifilm pressure mapping system along with a prescale calibration sheet and its cover for automatic calibration, as depicted in Figure 7.

**Figure 8:**
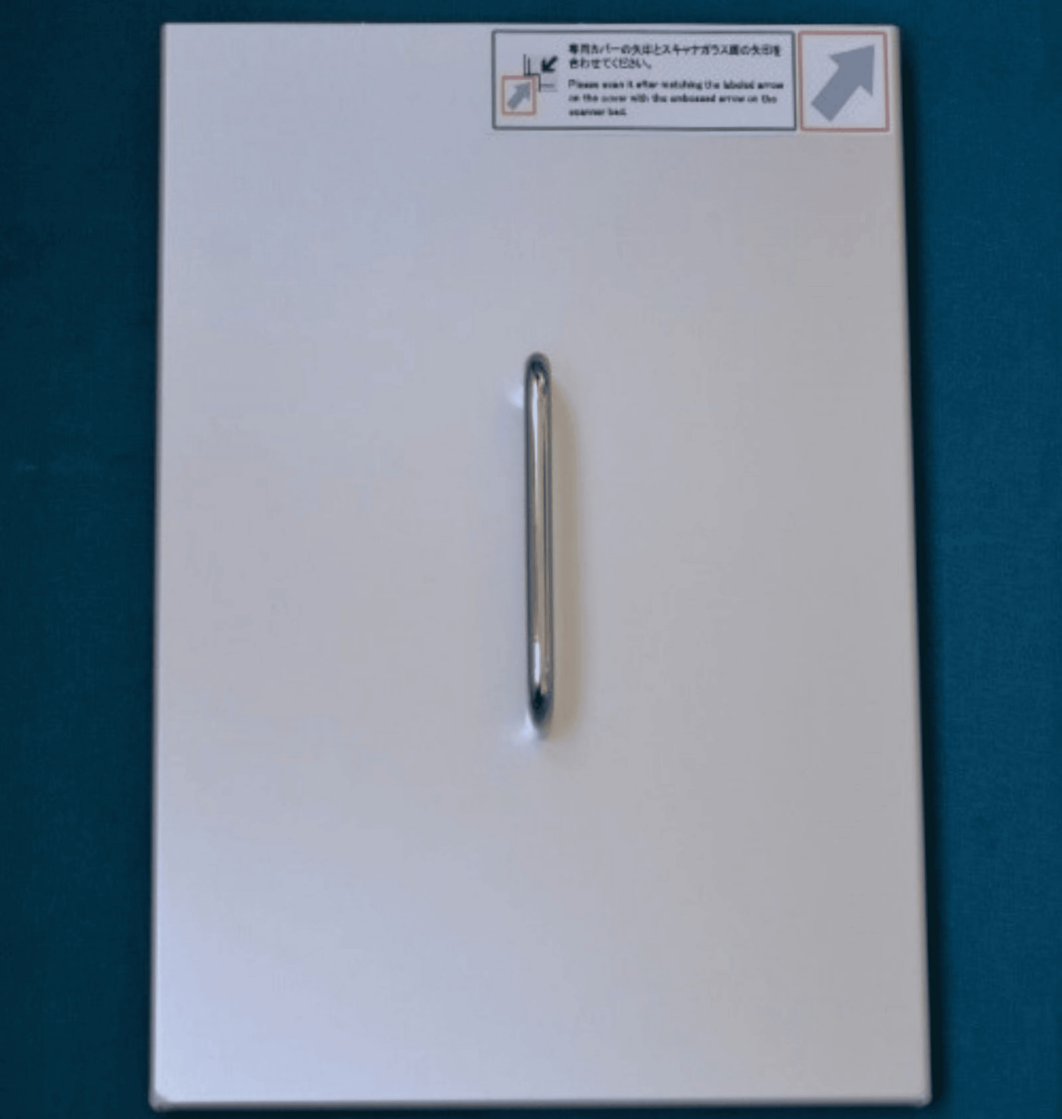
Metal disk for Epson V600 Perfection Scanner This is a metal cover used to cover the scanner surface while recording the reading. EPSON^®^ is a registered trademark, and Epson Perfection™ is the trademark of Seiko Epson Corporation (Suwa, Nagano, Japan).

Figure 8 illustrates the metal disk that shields the Epson scanner during the scanning of the pressure film.

Recording Pressure in Dental Patients

**Figure 9:**
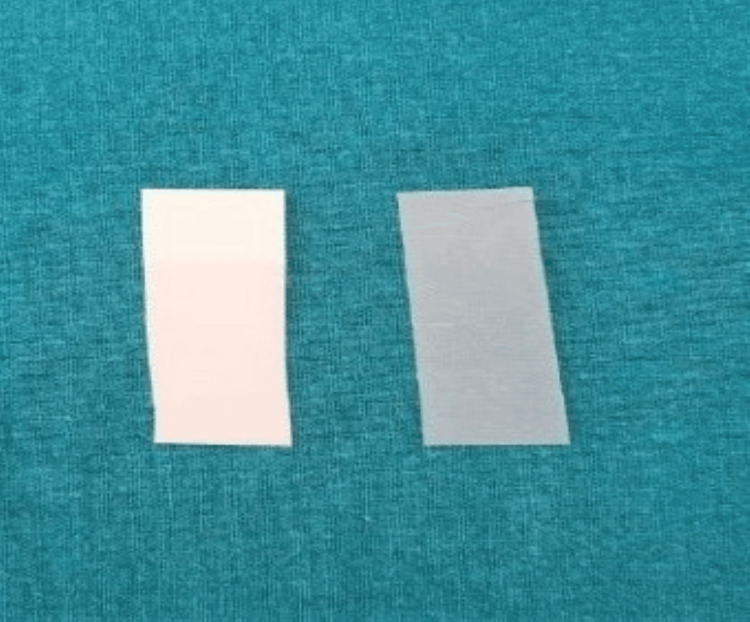
Fujifilm pressure-sensitive film without bite marks Film without any contact.

**Figure 10:**
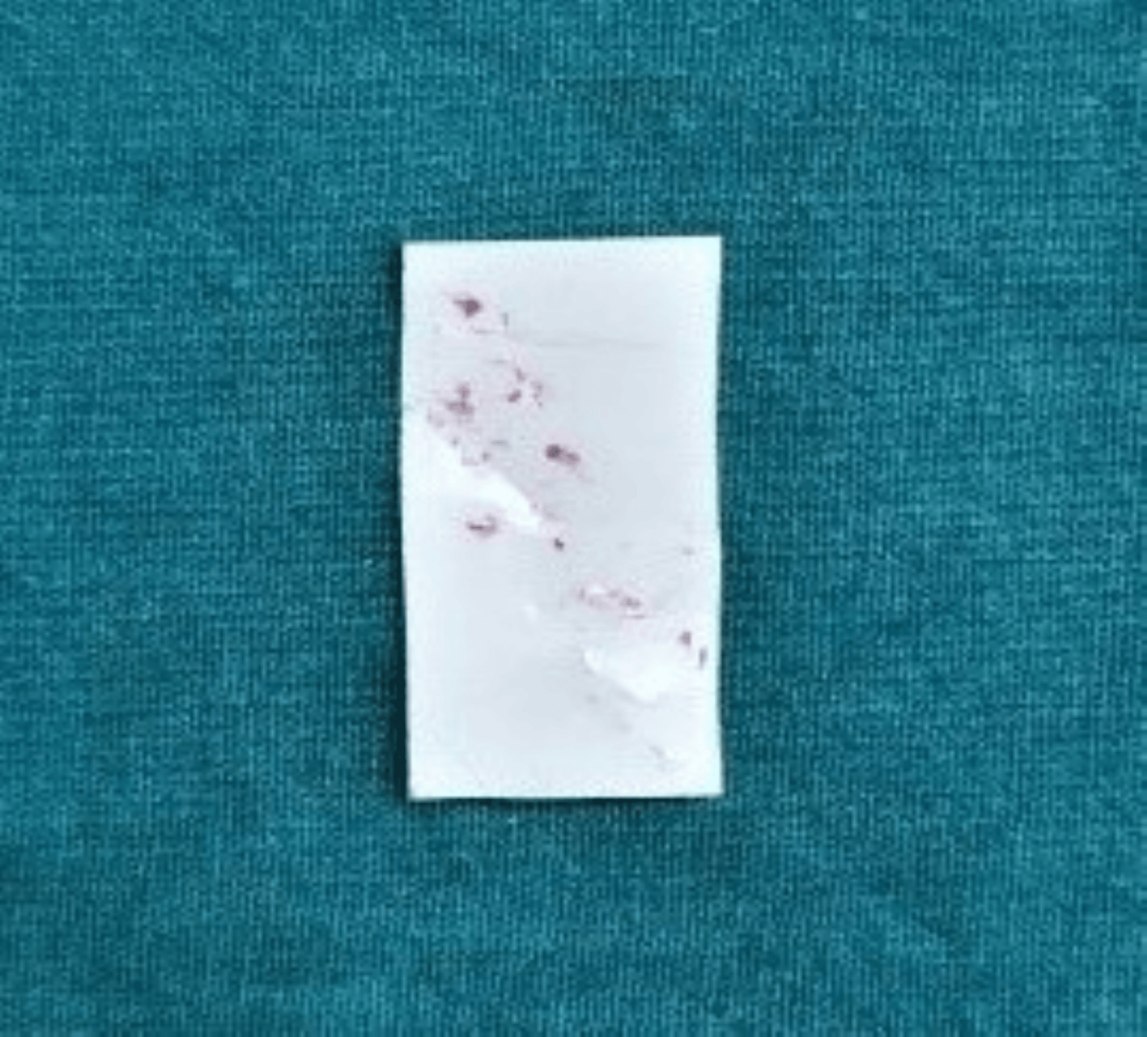
Fujifilm pressure-sensitive film with bite marks Film color changes after the bite is registered by the patient.

The pressure-sensitive film was positioned in the posterior area of the mouth with the rough surfaces facing each other. The patient was instructed to bite down for 30 seconds. As illustrated in Figures 9, 10, the film’s color changed based on tooth contact. Afterward, the film was taken out.

**Figure 11:**
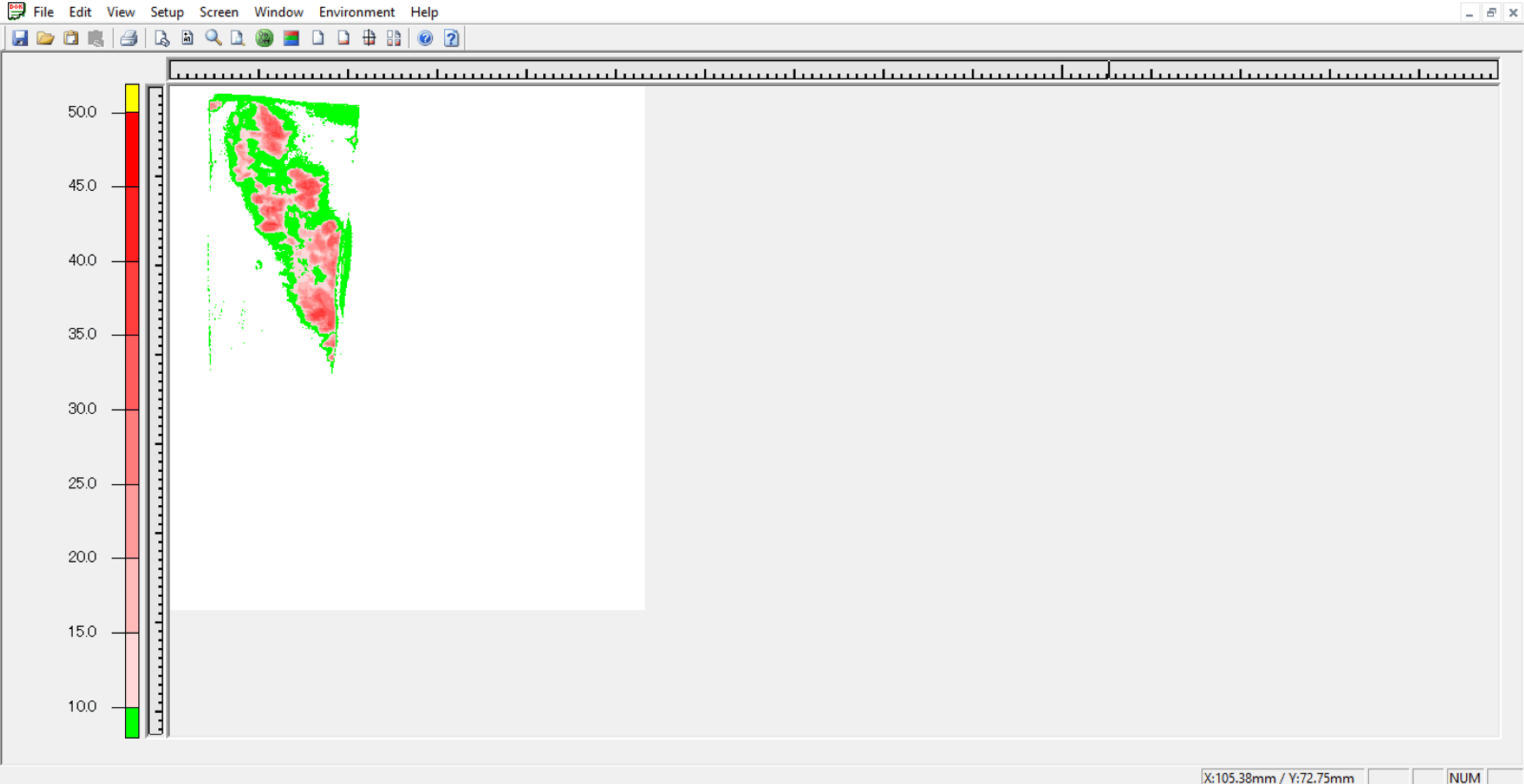
Image of bite recorded in FPD-8010E for Windows The FPD-8010E is a pressure image system that scans during pressurization and converts this into pressure values.

The scanner was first calibrated using the calibration sheet, and then the bite was scanned, with readings recorded as shown in Figure 11.

The bite was recorded at the time of insertion, and the reading was recorded. After one month of insertion, again the bite was recorded with the same process with both attachments.

Data Analysis

Data were analyzed for normal distribution using the Kolmogorov-Smirnov test and were found to have normal distribution. Therefore, a parametric test of significance was applied. Descriptive statistics were performed. Data were presented as mean and standard deviation. Intergroup comparison was done using an independent ‘t’-test. Intragroup comparison was done using a paired ‘t’-test. A p-value less than 0.5 was considered statistically significant. The effect size was chosen based on Cohen's guidelines, anticipating a substantial difference in the masticatory bite force between ball and flat attachments, ensuring the study is adequately powered to detect clinically meaningful differences. 

*Hypothesis* 

Null hypothesis: There is no significant difference between the masticatory bite force of implant-supported overdentures with ball and flat attachments.

Alternate hypothesis: There is a significant difference between the masticatory bite force of implant-supported overdentures with ball and flat attachments.

Hence, in this study, the alternate hypothesis was accepted.

Study Flowchart

**Figure 12:**
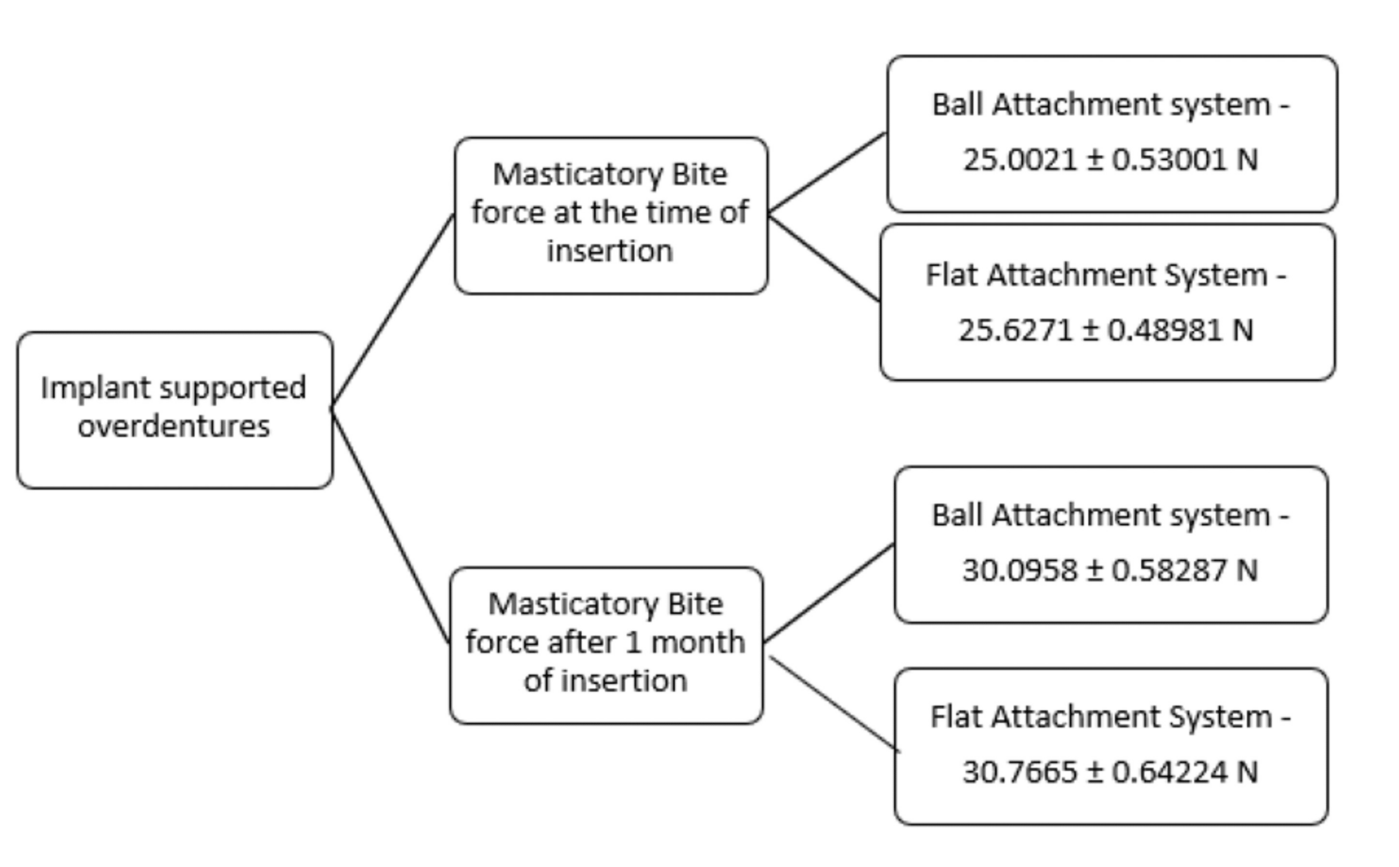
Study flowchart

Figure 12 shows the flowchart of the study.

Comparison of the baseline characteristics of both attachment systems is provided in Table 1. 

**Table 1:** Characteristics of the attachment systems

	Ball attachment	Flat attachment
Baseline characteristics	Convex ball-shaped component designed for rotational movement and a socket to receive it	Rectangular forms with distinct length, width, and height parameters for force transmission
Shape	Hemispherical or ball-shaped component that fits into a cup-like depression or socket	Flat, often rectangular or planar surface
Function	Rotational or circular movement, providing a large range of motion	Broad, stable contact area and serves as a fixed point for force application

## Results

The present study was done to compare the masticatory bite force in an implant-supported overdenture by using two different attachments. The attachments used were the ball attachment and the flat attachment. The bite force was registered in Newtons. The following results were obtained.

Table 2 shows the masticatory bite force of implant-supported overdentures in Group I at the time of denture insertion and after one month of denture insertion.

**Table 2:** Masticatory bite force of implant-supported overdentures in Group I at the time of denture insertion and after one month of denture insertion In Group I, the masticatory bite force of implant-supported overdenture with ball attachment was significantly greater after one month of denture insertion than at the time of denture insertion (25.6271 ± 0.48981 N vs 25.0021 ± 0.53001 N) (p-value <0.05). *p-Value <0.05 was considered statistically significant.

	Mean	Standard deviation	Difference in mean	t-Value	p-Value
At the time of denture insertion	25.0021	0.53001	-0.62500	-23.225	0.001*
After 1 month of denture insertion	25.6271	0.48981			

Table 3 shows the masticatory bite force of implant-supported overdentures in Group II at the time of denture insertion and after one month of denture insertion.

**Table 3:** Masticatory bite force of the implant-supported overdentures in Group II at the time of denture insertion and after one month of denture insertion In Group II, the masticatory bite force of implant-supported overdenture with flat attachment was significantly greater after one month of denture insertion than at the time of denture insertion (30.7665 ± 0.64224 N vs 30.0958 ± 0.58287 N) (p-value <0.05). *p-Value <0.05 was considered statistically significant.

	Mean	Standard deviation	Difference in mean	t-Value	p-Value
At the time of denture insertion	30.0958	0.58287	-14.814	-0.67063	0.001*
After 1 month of denture insertion	30.7665	0.64224			

Table 4 shows that the masticatory bite force for the implant-supported overdenture in Group II was significantly higher than in Group I at insertion (30.0958 ± 0.58287 vs 25.0021 ± 0.53001 N) (p < 0.05).

**Table 4:** Masticatory bite force (in Newtons) of the implant-supported overdenture with ball and flat attachments at the time of denture insertion Independent ‘t’-test. The masticatory bite force of implant-supported overdenture in Group II was significantly greater compared to that in Group I at the time of denture insertion (30.0958 ± 0.58287 vs 25.0021 ± 0.53001 N) (p-value <0.05). *p-Value <0.05 was considered statistically significant.

Group	Mean ± standard deviation	Difference in mean	t-Value	p-Value
Group I	25.0021 ± 0.53001	-5.09375	-18.288	0.001*
Group II	30.0958 ± 0.58287			

Additionally, Table 5 shows that even after one month, the bite force in Group II remained significantly greater than in Group I (30.7665 ± 0.64224 vs 25.6271 ± 0.48981 N) (p < 0.05).

**Table 5:** Masticatory bite force (in Newtons) of the implant-supported overdenture with ball and flat attachments after one month of denture insertion Independent ‘t’-test. The masticatory bite force of implant-supported overdenture in Group II was significantly higher compared to that in Group I (30.7665 ± 0.64224 vs 25.6271 ± 0.48981 N) (p-value < 0.05). *p-Value <0.05 was considered statistically significant.

Group	Mean ± standard deviation	Difference in mean	t-Value	p-Value
Group I	25.6271 ± 0.48981	-5.13938	-17.997	0.001*
Group II	30.7665 ± 0.64224			

## Discussion

In this study, the alternative hypothesis was accepted. Eight completely edentulous patients who had two mandibular implants were selected for the fabrication of implant-supported mandibular overdentures. The customary procedures for fabricating one maxillary and two mandibular dentures were followed. Subsequently, the mandibular dentures were converted into overdentures using ball and flat attachments via the pickup method. Masticatory bite force was measured with both attachments on the left and right sides at the time of denture insertion and again after one month using a bite force measuring device. The Fujifilm pressure distribution mapping system for prescale FPD-8010e (version 2.5) is a pressure imaging system that scans during pressurization and converts this into pressure values. This system includes software and a sheet for automatic calibration and utilizes the user’s computer as its processing engine. It provides a recorded bite in 3D software, which can also be enlarged at a particular point for reading.

Shah et al. [13] found that Nupai bite scanning has limited use for occlusal analysis due to its static nature; however, it proves beneficial in contexts where the amount of force, rather than its dynamics, is crucial. Isni et al. [14] recorded a total bite force of 1423 N using the Dental Prescale System, which was notably higher than the 256 N obtained with a unilateral bite force recorder. Nevertheless, the maximum bite force values from both methods were significantly correlated (r = 0.46, p < 0.05). Horibe et al. [15] indicated that reference values need to be established for occlusal force tests used in diagnosing oral hypofunction, both for the Prescale II without and with a pressure filter. Their findings imply that Prescale II can serve as a valid diagnostic tool for oral hypofunction. Wang et al. [16] concluded that the number of scans had no considerable impact on the four measurements analyzed. However, all measurements, except for contact area, were significantly influenced by scan delay; longer delays led to a greater increase in measurements.

In the current study, the masticatory bite force of the implant-supported overdenture with a ball attachment was significantly higher one month after denture insertion (25.6271 ± 0.48981 N) than the force recorded at the time of insertion (25.0021 ± 0.53001 N).

Furthermore, the masticatory bite force of the implant-supported overdenture with a flat attachment increased significantly after one month of denture insertion (30.7665 ± 0.64224 N) compared to the time of denture insertion (30.0958 ± 0.58287 N). This improvement may be due to the denture adapting to the patient’s mouth and the patient’s increased comfort with the denture. This finding aligns with a previous study by Pandey [17], which concluded that masticatory efficiency improved significantly during the second and third follow-ups compared to the first follow-up on both sides.

At the time of denture insertion, the masticatory bite force of the implant-supported overdenture in Group II (flat attachment) (30.0958 ± 0.58287 N) was significantly greater than that in Group I (ball attachment) (25.0021 ± 0.53001 N). Additionally, after one month of denture insertion, the masticatory bite force in Group II (30.7665 ± 0.64224 N) remained significantly higher than in Group I (25.6271 ± 0.48981 N). These findings are consistent with several studies, like Abdelfattah and Fahmi [18], which found that both ball/O-ring and locator attachment systems effectively improve the retention and stability of implant-retained mandibular overdentures, with the locator attachment showing superior initial stability.

However, the results of this study contrast with those of a study by Helmy and Kothayer [19], who found no statistically significant difference in the bite force and occlusal force distribution between the locator attachment and the ball attachment in retained mandibular overdentures.

Clinical implications of the study

Findings may guide clinicians in choosing between ball and locator attachments for implant-supported overdentures based on masticatory bite force. Overdentures based on masticatory bite force offer valuable insights into the biomechanical differences that impact retention, stability, and chewing efficiency. These differences have the potential to influence patient satisfaction and comfort with implant-supported prostheses. Consequently, understanding these factors could shape decisions in prosthodontic rehabilitation for edentulous patients.

Strength of the study

Direct ball vs. locator attachment comparison offers practical clinical insights. Masticatory bite force is a quantifiable metric relevant to prosthetic function. Bite force measurement reflects the functional aspect of implant-supported overdentures.

Limitations of the study

This study focused on two attachment systems, although various options are available for overdentures, including magnets, telescopic systems, bars, and clips. Analyzing the masticatory bite forces of these other attachment systems is essential. The Fujifilm prescale system can also assess the masticatory bite forces of different prostheses in both edentulous and dentulous patients. Geriatric patients typically express concerns about masticatory efficiency; thus, flat attachments may enhance masticatory bite force compared to ball attachments in implant-supported overdentures.

## Conclusions

A conventional complete denture is one of the most prevalent treatment modalities in the rehabilitation of a completely edentulous individual. A conventional complete denture can be a great discomfiture for edentulous individuals due to lack of stability, retention, social concern, i.e., slippage, unnatural appearance, and continued loss of bone leading to further instability of the denture, especially in a mandibular denture. Implant-supported prosthesis is a technique of enhancing the conventional denture retention, support, and stabilization. It can also be a practical type of therapy for satisfied denture wearers who want their prostheses to have extra stability. It has been documented that treatment with an implant-retained/-supported mandibular overdenture opposed by a conventional maxillary denture should be considered first.

Implant-supported overdentures offer a less invasive and more economical solution for mandibular edentulism compared to fixed prostheses. Different attachment systems are available commercially according to the needs and the conditions of patients. Some of the attachments are bar and clip attachments, stud attachment, ball attachment, and magnet attachment, out of which ball, flat, and magnet attachments are used most commonly because of their simple application compared to bar and clip attachments. In the present study, the masticatory bite force of implant-supported overdentures with different attachments was evaluated and compared between Group I (implant-supported overdenture with ball attachments) and Group II (implant-supported overdenture with flat attachments). It was found that an implant-supported overdenture with flat attachments has a greater masticatory bite force than an implant-supported overdenture with ball attachments. Additionally, the masticatory bite force increased one month post-insertion compared to the force at the time of placement.

## References

[1] (2017). The glossary of prosthodontics terms: Ninth edition. J Prosthet Dent.

[2] Wright PS (2006). Two implants for all edentulous mandibles. Br Dent J.

[3] Abdelhamid AM, Metwally NA, Imam MH (2016). The effect of two different attachments with implant retained mandibular overdentures on the masticatory function. J Dent Health Oral Disord Ther.

[4] Sharma AJ, Nagrath R, Lahori M (2017). A comparative evaluation of chewing efficiency, masticatory bite force, and patient satisfaction between conventional denture and implant-supported mandibular overdenture: An in vivo study. J Indian Prosthodont Soc.

[5] Chang M, Chronopoulos V, Mattheos N (2013). Impact of excessive occlusal load on successfully-osseointegrated dental implants: A literature review. J Investig Clin Dent.

[6] Melescanu Imre M, Marin M, Preoteasa E, Tancu AM, Preoteasa CT (2011). Two implant overdenture - The first alternative treatment for patients with complete edentulous mandible. J Med Life.

[7] Misch CE (2015). Dental Implant Prosthetics, 2nd ed. Mosby: Elsevier.

[8] Alsabeeha NHM, Payne AGT, Swain MV (2009). Attachment systems for mandibular two-implant overdentures: A review of in vitro investigations on retention and wear features. Int J Prosthodont.

[9] Oncescu Moraru AM, Preoteasa CT, Preoteasa E (2019). Masticatory function parameters in patients with removable dental prosthesis. J Med Life.

[10] Flanagan D (2017). Bite force and dental implant treatment: A short review. Med Devices (Auckl).

[11] Verma TP, Kumathalli KI, Jain V, Kumar R (2017). Bite force recording devices - A review. J Clin Diagn Res.

[12] Elsyad MA, Khairallah AS (2017). Chewing efficiency and maximum bite force with different attachment systems of implant overdentures: A crossover study. Clin Oral Implants Res.

[13] Shah P, Anehosur G, Nadiger R, Lekha K (2015). A study to compare sensitivity of nupai bite scan to T scan system. Indian J Stomatol.

[14] Isni KHK, Ja-Hea Y, Young-Sook Y, Isni KB (2006). Comparison of bite force with dental prescale and unilateral bite force recorder in healthy subjects. J Korean Acad Prosthodont.

[15] Horibe Y, Matsuo K, Ikebe K, Minakuchi S, Sato Y, Sakurai K, Ueda T (2022). Relationship between two pressure-sensitive films for testing reduced occlusal force in diagnostic criteria for oral hypofunction. Gerodontology.

[16] Wang TM, Chang YH, Yang TC, Lin LD (2022). Effect of scan delay on measurements of an occlusal pressure sensitive film: An in-vitro study. J Dent Sci.

[17] Pandey KK (2020). A study to evaluate the role of complete dentures in improving the chewing efficiency of edentulous patients. J Indian Prosthodont Soc.

[18] Abdelfattah MY, Fahmi MK (2020). Evaluation of two different attachment systems used with mandibular implant‑retained overdenture. J Dent Impl.

[19] Helmy MA, Kothayer M (2019). Effect of two different overdenture attachments on the biting force and occlusal force distribution. Egyptian Dent J.

